# A Facer for Christian Science

**Published:** 1901-05

**Authors:** 


### A FACER FOR CHRISTIAN SCIENCE.

THE Christian Scientists of Atlanta have suffered a severe if entirely merited rebuff at the hands of the Hon. J. H. Lumpkin, Judge of the Superior Court. In denying the application for charter for the Atlanta Institute of Christian Science, Judge Lumpkin rendered the decision that Christian Science was within the definition of practice of medicine, and that its advocates were not legally qualified to practice. He stated that "the constitution of Georgia guaranteed freedom of conscience and religious worship, but that it nowhere guarantees the right to have certain religious ideas incorporated for business purposes, especially where there is strict legislation on the subject."

He held that "the law touching the incorporation of churches shows plainly that they are incorporated 'not for the purpose of trade and profit, but for the purpose of promoting the design of such institution,' and that it then provides that the Superior Court 'may grant such person or persons and their legal successors such corporate powers as may be suitable and not inconsistent with the laws of this State nor violative of private rights.'"

The decision states that the refusal of the charter has nothing to do with any question of religious belief or freedom of conscience.

Nothing could be more just than Judge Lumpkin's decision. In fact the law is so full and explicit on this subject that it is inconceivable that any other decision could have been or would be rendered. We quote from the Code of Georgia, section 1409 (b): "*Practice of Medicine Defined.* For the purposes of this chapter the words 'practice medicine' shall mean to suggest, recommend, prescribe or direct for the use of any person any drug, medicine, appliance, apparatus or other agency, whether material or not material, for the cure, relief or palliation of any ailment or disease of the mind or body, or for the cure or relief of any wound, fracture or other bodily injury or any deformity after having received, or with the intent of receiving therefor, either directly or indirectly, any *bonus*, gift or compensation."

There can be no question as to the illegality of Christian Science or any other irregular and aberrant creed which has for it avowed or implied object the treatment of disease. This much is clear, but what is not clear is for what reason are these irregulars allowed to flaunt their brazen effrontery in the faces of the officers of the law and to ply their proscribed avocation in open violation of legal ordinances? Our contention has always been that our laws were satisfactory and sufficient, but that they were openly ignored, contemned and set to scorn and defiance.

The insidious hold of Christian Science is largely owing to its alleged divine origin and its spurious claim to reverential consideration. When attacked, its advocates cry "blasphemy," and find an unmerited protection within the horns of the altar. There has never been such a monstrous perversion of the Word since the days of the false prophets. The system is in itself the essence of blasphemy. It recoils upon itself. It is a god slain on his own altar, if there is anything godlike about it. There need be no scruple over assailing this most incoherent and absurd aberration. It is entitled to no respect or consideration at the hands of the reverential or any other. Even if it were otherwise, there is the example of Christ upsetting the tables of the money-changers when God's house was changed into a house of merchandise.

